# *Ullucus tuberosus*: A Review of Its Biology, Nutritional Profile, Phytochemistry, and Food Industry Applications

**DOI:** 10.3390/foods15152705

**Published:** 2026-07-31

**Authors:** Anabel Bolaños-Narciso, Emerson Asto-Rodriguez, Luz María Paucar-Menacho, Williams Esteward Castillo-Martinez

**Affiliations:** 1 Programa de Doctorado en Ingeniería Agroindustrial, Mención Transformación Avanzada de Granos y Tubérculos Andinos, Universidad Nacional del Santa, Nuevo Chimbote 02712, Peru; 2025818023@uns.edu.pe (A.B.-N.); 2025818021@uns.edu.pe (E.A.-R.); 2 Departamento Académico de Agroindustria y Agronomía, Facultad de Ingeniería, Universidad Nacional del Santa, Nuevo Chimbote 02712, Peru; luzpaucar@uns.edu.pe

**Keywords:** *Ullucus tuberosus*, betalains, Andean tubers, functional properties of starch, clean-label colourants, forgotten crop

## Abstract

Ulluco (*Ullucus tuberosus*), the second most economically important Andean tuber after potato, has been cultivated for over 5500 years. Despite its resistance to frost, drought, and high altitudes, as well as its biochemically complex profile, ulluco remains underrepresented in the scientific literature and absent from international markets. This comparative review synthesises the evidence available up to November 2025 regarding its biology, nutritional profile, phytochemistry, and food industry applications relative to four other Andean tubers: oca (*Oxalis tuberosa*), mashua (*Tropaeolum tuberosum*), native potato (*Solanum tuberosum* L.), and yacon (*Smallanthus sonchifolius*). The main comparative findings are as follows: First, ulluco is the only Andean tuber that biosynthesises betalains instead of anthocyanins, producing up to 32 distinct compounds that are stable across a pH range of 3–7, a chromatic stability advantage over anthocyanin-based pigments from mashua, oca, and native potato, which suggests its potential as a source for the development of clean-label natural colourants. Second, among the five species evaluated, ulluco protein content across different morphotypes ranges from 5.60 to 15.7 g/100 g dry weight, ranking within the upper range of values reported for mashua and oca, and comparable to or higher than the upper ranges reported for native potato and yacon. This variability reflects the genotypic and ecogeographical diversity of the evaluated accessions and is not a methodological inconsistency, with 20% of varieties exceeding 10 g/100 g and providing six essential amino acids, supporting genetic improvement programmes aimed at nutritional security. Third, ulluco starch exhibits a low gelatinisation temperature (56–60 °C), a high amylose content (24–36%), and pseudoplastic behaviour, properties comparable or superior to potato starch for specific applications, with technical viability validated at a laboratory scale in applications including biodegradable films, food 3D printing inks, and dehydrated snacks. Unlike yacon, which accumulates fructooligosaccharides and lacks starch, ulluco aligns with oca, mashua, and potato as a starch-accumulating species. Critical gaps identified include the absence of clinical evidence on betalain bioavailability, the lack of a complete nuclear genome assembly, and the non-existence of standardised processing methods for industrial scaling. A coordinated agenda that integrates clean seed programmes, pilot-scale validation, and genomic breeding is essential for the bioeconomy of the 21st century to transform ulluco into a high-value ingredient.

## 1. Introduction

Ulluco (*Ullucus tuberosus*), belonging to the Basellaceae family, is an important Andean tuber crop with a long history of cultivation [1,2,3]. Its domestication in the central Andes, in the territories now occupied by Peru and Bolivia, dates back approximately 5500 years, with archaeological evidence documented in phytomorphic representations of the Moche culture and other pre-Hispanic periods dated between 2250 and 2050 B.C. [2,4,5].

Historically, ulluco has been the second most important tuber economically and nutritionally in the Andean region, surpassed only by the potato (*Solanum tuberosum* L.), with a cultivation area extending from Venezuela to northwestern Argentina [2,3,6,7,8]. Despite this millennial character and its recognition as a valuable Andean crop in international markets such as New Zealand, its expansion outside South America has been limited by photoperiod and climatic adaptability barriers [1,7,9,10].

In the 21st century, ulluco is considered on the global scientific agenda as a “forgotten crop” or “species of opportunity for development” [11]. Its current relevance lies in its remarkable resilience, as it thrives in marginal agroecosystems with poor soils, recurrent droughts, and altitudes above 4000 m a.s.l., where few crops survive [9,12]. However, this resilience faces projected threats under climate change scenarios.

In parallel, the industry has identified ulluco as a unique source of betalains, pigments with high antioxidant capacity absent in other Andean tubers, which produce anthocyanins, positioning it as a functional ingredient to combat chronic diseases and cellular ageing [1,6,7,13,14].

This comparative review synthesises the scientific evidence available up to November 2025 on *Ullucus tuberosus* relative to four other Andean tubers: native potato (*Solanum tuberosum* L.), oca (*Oxalis tuberosa*), mashua (*Tropaeolum tuberosum*), and yacon (*Smallanthus sonchifolius*). The analysis is organised into five thematic axes: (i) biology, ecotype diversity, and genomic resources; (ii) nutritional profile and bioactive compounds, with emphasis on protein variability and the uniqueness of betalains; (iii) processing and preservation technologies; (iv) applications in the modern food industry; (v) a critical analysis of research gaps, projections under climate change, and a roadmap for sustainable industrialisation. Governance considerations aimed at ensuring that the valorisation of the crop benefits the Andean communities of origin are also addressed.

## 2. Methodology

This review is based on a bibliographic search aimed at integrating the available evidence on *U. tuberosus* physiology, biochemistry, processing technology, and industrial potential. The units of analysis included original primary studies of a descriptive and experimental nature, technical reports from reference organisations, and agricultural research-related theses.

The sources were identified in high-impact databases such as Scopus, Web of Science, ScienceDirect, PubMed, SciELO, and Google Scholar. The search terms used were as follows: “*Ullucus tuberosus*”, “ulluco”, “betalains”, “Andean tuber starch”, “bioactive compounds”, and “food industrial applications”.

Studies on other Andean tubers were also included, such as oca (*Oxalis tuberosa*), mashua (*Tropaeolum tuberosum*), and native potato (*S. tuberosum*, which encompasses the Andean cultivar groups historically recognised as *S. tuberosum* ssp. *andigenum* and *S. phureja*) through specific searches using their scientific and common names to construct a nutritional, phytochemical, and industrial reference framework. Yacon (*Smallanthus sonchifolius*) was incorporated into this comparative framework because of its appearance in retrieved studies on ulluco or other tubers from the region, without constituting an independent search objective.

## 3. Biology and Varieties

### 3.1. Description of the Main Ecotypes

Ulluco constitutes the only species of the monotypic genus *Ullucus* (family Basellaceae) [1,15]. This perennial herbaceous plant is the second most economically important tuber in the Andean region, surpassed only by the potato (*S. tuberosum*) [6,16,17], with a hypothetical biogeographical origin in the central area of Peru and Bolivia, or alternatively in the Cundiboyacense savanna of Colombia [18].

**Figure 1:**
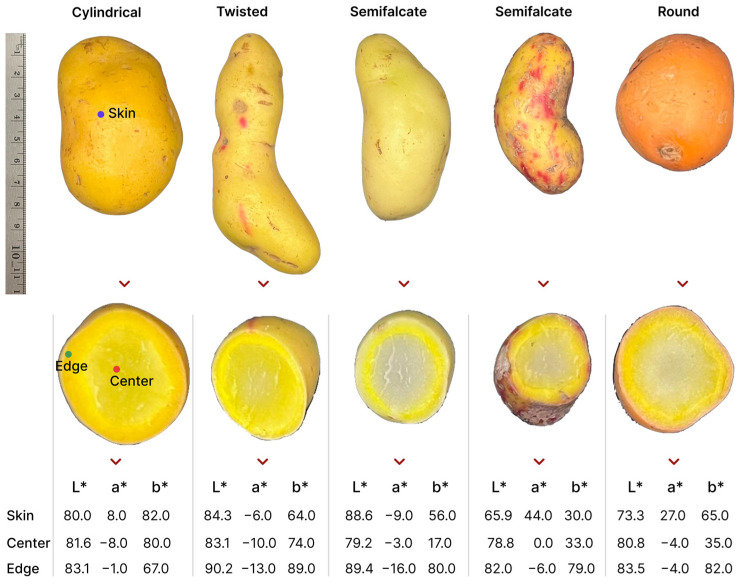
Morphological and colorimetric characterisation of *Ullucus tuberosus* tubers. The upper row displays the diverse tuber shapes (cylindrical, twisted, semifalcate, and round) with a reference scale on the left. The middle row shows cross-sections highlighting internal flesh distribution. The lower table presents the CIELAB colour coordinates (L*, a*, b*) measured at three distinct anatomical zones: skin, centre, and edge.

Morphologically, the plant exhibits succulent and mucilaginous stems with either erect or prostrate growth habits, reaching heights of 20 to 80 cm [15,19]. The tuber forms at the end of adventitious roots or stolons and exhibits great phenotypic diversity: spherical, cylindrical, falcate or twisted shapes, with a waxy, bright skin in shades ranging from white and yellow to orange, red, magenta, and purple [4,9,20,21]. This geometric variation, together with the internal spatial distribution of colour among the different tuber tissues, is detailed in Figure 1, which also illustrates the colorimetric characteristics of different ulluco varieties and their tissues.

Furthermore, this pigmentation is unique among Andean tubers, as it is due to betalains (betacyanins and betaxanthins) synthesised from tyrosine, instead of anthocyanins [1,6,22]. The infraspecific diversity of ulluco is classified into two groups according to growth habit. The creeping type, characteristic of the northern Andes (Colombia and Venezuela), features long stems (up to 1.5 m), small leaves, and elongated red-purple tubers, possibly similar to the wild forms [9,12,18,19]. The erect type, which is predominant in Peru and Bolivia, produces compact plants with deep green leaves and greater variability in tuber colouration [9,12].

**Figure 2:**
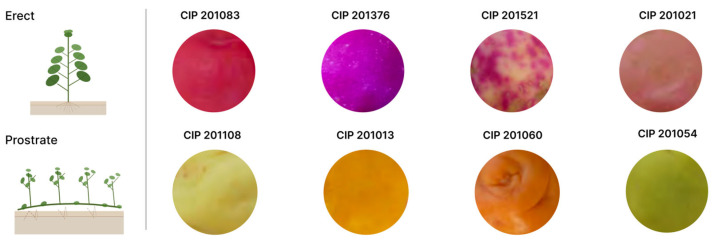
Phenotypic variation in *Ullucus tuberosus* ecotypes. Illustration of growth habits (erect vs. prostrate) and examples of tuber skin pigmentation from the CIP collection. The displayed skin varieties highlight a subset of the wider spectrum of intermediate shades.

Regarding ploidy, most cultivars are diploid (2*n* = 24), although triploids (2*n* = 36) and tetraploids exist, which are associated with a certain sterility or low sexual seed production [9,19,23]. In Peru, ecotypes such as *Chuqchan lisa* (elongated), *Q’ello chuqcha* (yellow), *Muru lisa* (pink), *Yuraq lisa* (white), and *Puka lisa* (reddish) are commercially recognised [9,10,12]. Wild relatives (*U. tuberosus* subsp. *aborigineus*) also exist, with long climbing stems and small, bitter tubers [9,12]. The plant growth habits and the breadth of this chromatic diversity, resulting from centuries of Andean selection, are illustrated in Figure 2, based on accessions from the International Potato Center (CIP).

Archaeobotanical evidence indicates a long history of ulluco use in the Andean region. A study of plant remains from the Taraco Peninsula, Bolivia, covering the period from 1500 BCE to 1000 CE, examined carbonised tissues using scanning electron microscopy [24]. The findings suggest that ulluco, together with potato, oca, and mashua, was present in the diet of early settlements (1400–700 BCE) in the Titicaca Basin [24]. Ulluco has a very thin skin and specific internal tissue characteristics that distinguish it from other tubers [24].

### 3.2. Factors Influencing Cultivation Quality

The crop grows optimally between 2800 and 4000 m a.s.l., with an ideal range of 3000–3600 m, temperatures between 8 and 14 °C, and rainfall of 600–1000 mm [9,12]. Its sensitivity to photoperiod demands short days for proper tuberisation; under long day conditions, it can inhibit tuber formation or alter their morphology, although the colour tends to remain stable [23]. Ulluco is tolerant to frost and drought, allowing it to thrive where other crops, such as maize, do not survive [3,12,18].

It prefers light-textured soils, rich in organic matter, and with a slightly acidic pH (5.5–6.5). Compacted soils inhibit tuber thickening and compromise their commercial value. Although it tolerates poor soils, it responds positively to organic fertilisation [12,25].

Propagation is predominantly vegetative using seed tubers [9], which favours the accumulation of viruses, such as *Ulluco mosaic virus* and other potyviruses, that degenerate the material and limit productivity [9,26]. The vegetative cycle lasts between 7 and 9 months depending on the altitude and variety [3,12,19]. Due to its high water content (80 to 85%) and thin skin, ulluco is highly perishable; exposure to light can cause the tubers to green and affect their commercial quality [10,12].

### 3.3. Comparative Analysis: Ulluco Versus Other Andean Tubers

Table 1 presents a comparative analysis that highlights ulluco’s distinctive nutritional and functional profile. While oca, mashua, native potato, and yacon offer valuable reference points, ulluco emerges with a combination of traits that make it exceptionally suitable for specific industrial and food applications [3,27].

The most notable differentiator of ulluco compared to other Andean tubers is its pigmentation system, which is based on betalains instead of anthocyanins. This positions it phylogenetically alongside the order Caryophyllales and confers critical technological advantages for food applications [1,6,22]. Section 4.4 provides a detailed analysis of this biochemical singularity and its industrial implications.

Regarding its protein content, ulluco ranks among the highest of the five species, presenting a range of 5.60–15.7 g/100 g on a dry basis [10,12,44]. This places it alongside mashua and the upper percentile of oca [12], significantly exceeding the typical values reported for native potato and yacon [3,25]. The variability observed within this range, documented across multiple accessions and ecotypes, indicates very significant intrinsic genetic diversity that could be strategically leveraged for biofortification programmes without recourse to external genetic modification. This aspect is relevant to food security and nutrition strategies in the high Andean regions, where ulluco serves as a staple food [2,25].

From a carbohydrate matrix perspective, ulluco aligns with oca, mashua, and native potato as a starch-accumulating species, reaching a pure starch content of up to 70.5% on a dry basis [10,27,41]. This profile places it in a taxonomic and agroindustrial category fundamentally different from yacon, which stores FOS and lacks starch [3,38]. Furthermore, the presence of mucilages, which are hydrocolloid polysaccharides with notable rheological properties, further distinguishes ulluco [12,20]. These functional compounds pave the way for advanced applications, such as the development of biofilms or biodegradable films, as well as their use as thickening and texturising agents in jellies or preserves [27,45]. These technological applications are less feasible in other species due to their divergent polysaccharide profiles [3].

**Figure 3:**
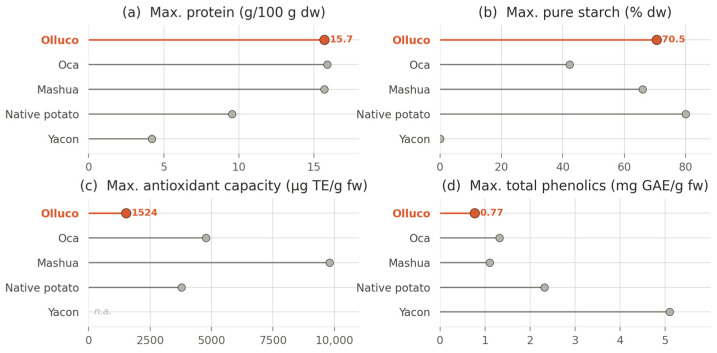
Comparative nutritional profile of *Ullucus tuberosus* (ulluco) and four Andean tubers: yacon (*Smallanthus sonchifolius*), native potato (*Solanum tuberosum* L.), mashua (*Tropaeolum tuberosum*), and oca (*Oxalis tuberosa*). Maximum reported values are shown for each parameter on independent scales. dw, dry weight; fw, fresh weight; TE, Trolox equivalents; GAE, gallic acid equivalents. n.a., not available.

Ulluco’s antioxidant capacity, though significant, occupies a moderate position within the Andean tuber family [6]. Its ABTS radical inhibition values range from 483 to 1524 µg of Trolox equivalents (TE) per gram of fresh weight, a metric that is lower than the peaks of activity documented in mashua and the upper ranges of oca and native potato [6,22]. However, this parameter should not be interpreted in isolation as a limitation. Ulluco’s true industrial potential lies not in competing exclusively on antioxidant capacity metrics, but in the synergy of its bioactive pigments (betalains), its appreciable protein content, and its mucilaginous polysaccharides. This is a phytochemical and rheological profile that no other species in this comparative matrix replicates in its entirety [2,3,22], as shown in Figure 3.

### 3.4. Genomics, Phylogeny, and Genetic Resources

Genomic knowledge of *U. tuberosus* is essential for tracing its domestication routes, accelerating its breeding, and ensuring plant material’s health status in a globalised market. This section reviews the genomic and transcriptomic knowledge of the crop, the molecular bases of its most distinctive metabolic pathway, and the molecular tools available for its conservation and traceability.

#### 3.4.1. Current Status of Genomic and Transcriptomic Sequencing

The development of the complete nuclear genome for *U. tuberosus* is in less advanced stages than that of other Andean starchy crops, such as *S. tuberosum*. Nonetheless, the application of modern genomic tools has been fundamental in resolving critical authentication and phytosanitary problems. At a taxonomic level, the use of DNA barcoding through the sequencing of chloroplast genes, such as ribulose-1,5-bisphosphate carboxylase (*rbcL*) and maturase K (*matK*), has allowed for the 100% identity validation of samples with reference sequences deposited in GenBank [13]. This precision is a necessary tool to guarantee traceability and prevent the adulteration of the raw material in the development of functional ingredients.

High-throughput sequencing (HTS) has significantly improved pathogen diagnosis in *U. tuberosus* germplasm. Using total RNA with ribosomal RNA depletion, combined with *de novo* assembly algorithms such as Trinity, researchers have reconstructed complete viral genomes and identified multiple previously unknown viruses [46]. Historically, phytosanitary certification relied on serological tests such as Enzyme-Linked Immunosorbent Assay (ELISA), which often produced false-positive results due to cross-reactivity with other pathogens [46]. However, the resolution provided by HTS demonstrated that isolates erroneously diagnosed as *Andean Potato Latent Virus* (APLV) actually corresponded to two novel species of the genus *Tymovirus*, formally named *Ullucus tymovirus 1* and *Ullucus tymovirus 2*, which show merely 63% to 66% genomic identity with APLV [46]. This capacity of transcriptomics to elucidate the true virome of the crop is now known to include species and strains exclusive to the genera *Potexvirus*, *Tobamovirus*, *Potyvirus*, and *Polerovirus* [46]. Despite these biotechnological advances, the gap in complete nuclear genome sequencing remains a significant limitation for mapping genes associated with critical agronomic factors, such as tuberisation under a long photoperiod [47].

#### 3.4.2. Molecular Basis of Betalain Biosynthesis

**Figure 4:**
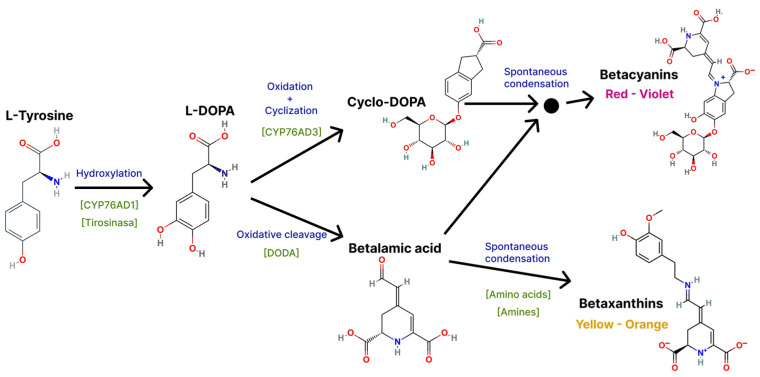
Biosynthetic pathway of betalains in Caryophyllales. L-tyrosine is converted to L-DOPA by CYP76AD enzymes. Subsequent reactions produce cyclo-DOPA and betalamic acid, whose spontaneous condensation leads to the formation of betacyanins and betaxanthins.

*U. tuberosus* is the only Andean tuber that synthesises betalains instead of anthocyanins [6,15,17]. These nitrogenous, water-soluble pigments derive from L-tyrosine. As shown in Figure 4, CYP76AD enzymes convert tyrosine to L-DOPA, from which two intermediates emerge: cyclo-DOPA and betalamic acid. Condensation of betalamic acid with cyclo-DOPA yields betacyanins (red to magenta), whereas condensation with free amino acids or amines yields betaxanthins (yellow to orange) [15].

Liquid chromatography coupled with diode array detection and electrospray ionisation tandem mass spectrometry (LC-DAD-ESI-MS/MS) has revealed up to 32 betalain variants in ulluco (20 betaxanthins and 12 betacyanins), including rare chemotaxonomic markers such as arginine-betaxanthin, glycine-betaxanthin (portulacaxanthin III), gomphrenin III, and isogomphrenin III [1,15]. Ploidy also influences pigment accumulation: oryzalin-induced tetraploid lines exhibit higher betacyanin concentrations than their diploid counterparts [1]. Table 2 lists the complete set of 32 betalains identified in ulluco, grouped by structural class.

#### 3.4.3. Molecular Markers for Conservation and Traceability

The genetic characterisation of ulluco has transitioned from morphological taxonomy to the use of highly reproducible molecular markers for managing germplasm collections, such as the International Potato Centre (CIP) collection, which exceeds 544 accessions [48]. Inter-Simple Sequence Repeat (ISSR) markers have revealed that, despite millennia of vegetative propagation, ulluco maintains a considerable genotypic diversity (46.8% polymorphism) with low genetic redundancy in extensive collections (1.6%) [49]. This relict diversity suggests the occurrence of sexual reproduction events in the evolutionary past and the accumulation of somatic mutations preserved by Andean farmers [49]. ISSRs have also served to validate medium-term in vitro conservation protocols, such as slow growth at 17 °C with mannitol, confirming DNA stability and the absence of somaclonal variation in the regenerated microshoots [50].

The complementary use of Random Amplified Polymorphic DNA (RAPD) markers and isozymes has revealed the spatial structure of the crop. In the Colombian Andes, two differentiated genetic pools (northeast and southwest) were identified, separated by geographical and linguistic barriers. Semi-domesticated forms were found in the northeastern region that combine wild traits with domesticated characteristics. This finding suggests that the primary forms migrated from the central Andes and that the Colombian populations completed independent domestication phases [19]. At the proteomic level, Sodium Dodecyl Sulfate Polyacrylamide Gel Electrophoresis (SDS-PAGE) has allowed for the typification of diversity through the profile of storage proteins. Consistent patterns in low-molecular-weight proteins, especially the bands at 14 and 24 kDa, serve as a stable biochemical fingerprint. Their combination with ploidy analysis, which includes diploids (2*n* = 24), triploids (2*n* = 36), and tetraploids (2*n* = 48), facilitates the estimation of genetic distances and phylogenetic relationships [2,19]. This integration of molecular and protein markers is useful for mitigating genetic erosion and ensuring the authenticity of local varieties with nutritional value [2,49].

#### 3.4.4. Bioprospecting and Food Sovereignty

Ulluco constitutes a strategic resource for food sovereignty in Andean highland agroecosystems. Its continuous cultivation by indigenous Quechua, Aymara, and Muisca communities has sustained semi-domesticated forms and numerous morphotypes as an adaptation to extreme climatic conditions [8,10,18,19]. However, the crop has suffered marginalisation, partly due to the stigma of being considered a subsistence food. The advancement of monoculture, changes in dietary habits, and rural–urban migration have increased its risk of genetic erosion [11,50].

The management of this agrobiodiversity requires combining ex situ and in situ conservation strategies. At the ex situ level, the CIP maintains over 544 accessions in vitro [48]. Cryopreservation techniques using droplet vitrification with cryoprotective solutions (PVS2) and medium-term conservation with mannitol at 17 °C have been implemented [48,50]. In situ conservation relies on subsistence farmers. Traditional agrobiodiversity fairs and the exchange of seed tubers act as genetic flow mechanisms that contribute to cultural resilience and mitigate germplasm homogenisation [18,49].

Bioprospecting has focused on characterising secondary metabolites with value for the nutraceutical, cosmetic, and pharmacological industries. Tuberocides, triterpenoid saponins with hypoglycaemic activity, and a singular betalain profile including arginine-betaxanthin, portulacaxanthin III, and gomphrenin III have been identified. These compounds position ulluco as a potential source of functional ingredients [1,6,7,15,51]. The insertion of ulluco into global markets requires the implementation of Access and Benefit-Sharing (ABS) protocols under the Nagoya Protocol, so that the commercialisation of its components provides a return to the communities of origin. The promotion of farmer associations, fair trade, and integration into value chains are fundamental tools for protecting Andean biocultural heritage [11].

#### 3.4.5. In Vitro Propagation and Phytosanitary Cleanup

The vegetative propagation of ulluco through seed tubers, while maintaining desirable agronomic traits, also facilitates the accumulation of viral pathogens that can reduce productivity over successive generations [9,26]. A micropropagation protocol has been developed for an Amazonian genotype using plant growth regulators to control the different stages of development [52]. The type and concentration of these regulators influence tissue regeneration, shoot proliferation, and rooting. Kinetin at a specific concentration achieved full tissue regeneration and promoted shoot elongation, whereas 6-benzylaminopurine produced the highest shoot proliferation [52]. For rooting, auxin 1-naphthaleneacetic acid gave the greatest root length, whereas indole-3-butyric acid produced a higher number of roots. Plantlets showed full survival during transfer to greenhouse conditions [52]. This study was conducted on a single genotype, and the response of other Andean morphotypes to this protocol has not been evaluated.

## 4. Nutritional Profile and Bioactive Compounds

The nutritional profile of *U. tuberosus* positions it as a genetic resource of notable value, boasting a density of nutrients and bioactive compounds that compares favourably with other Andean species in several parameters, as shown in Table 1 [7,22]. Its chemical composition is notably hydrated, with moisture levels ranging from 70% to 92%, which directly influences its texture and gastronomic versatility [7,10,12,20].

### 4.1. Macronutrients: Starch and Protein Profile

Starch is the predominant component of dry matter, accounting for approximately 65% of the dry weight [27,45], with extraction yields ranging from 43% to 65% and an amylose content of 23.9% to 36% [28,36,45]. This high proportion of amylose imparts notable thermal stability, low water vapour permeability, and excellent properties for the formation of bioplastics or 3D printing matrices [36,44,45]. The granules exhibit ellipsoidal and oval morphologies with high birefringence and show natural resistance to mechanical and thermal degradation superior to that of commercial potato starch [31,36,53]. The in vitro digestibility rate is greater than 80%, reaching up to 94% in certain morphotypes [6,7,35].

The free-sugar fraction of *U. tuberosus* is dominated by glucose (13.18%), followed by fructose (11.13%) and sucrose (6.08%) on a dry-weight basis [6], a composition that favours rapid assimilation and accounts for the mild natural sweetness noted in sensory evaluations. Total carbohydrates range from 73.5% to 84.2% of dry weight [4], whereas lipids comprise a minor fraction, between 0.1% and 1.4% [34]. This combination of high carbohydrate density and negligible fat reflects the role of the tuber as an underground storage organ optimised for energy accumulation rather than lipid reserve, positioning *U. tuberosus* as a candidate ingredient for low-fat, energy-dense formulations.

The tuber also contains a substantial mucilage fraction, a high-molecular-weight carbohydrate polymer composed of water, pectins, sugars, and organic acids. Biologically, this mucilage is thought to confer resistance to drought and frost stress in the high-altitude Andean environment [12], while technologically it enhances water-holding capacity and swelling power, properties exploited as thickening and gelling agents in food formulations.

*U. tuberosus* protein content ranges from 5.60 to 15.7 g/100 g on a dry weight basis. This range reflects data from two different studies. The upper value (15.7 g/100 g) was reported in an evaluation of five Andean cultivars [34]. The lower value (5.60 g/100 g) and the detailed distribution were obtained from a study of 50 varieties from the INIA germplasm bank, which found protein levels ranging from 5.60 to 11.55 g/100 g [2]. Both studies used the Kjeldahl method with a conversion factor of 6.25.

Three factors explain this variation. The first is genetic diversity. Electrophoretic analysis shows that while some proteins (14 and 24 kDa) are similar across varieties, others (33–80 kDa) differ [2]. A study that grew several ulluco cultivars in New Zealand under the same conditions found that some varieties had higher protein contents (12.19% and 11.83%) than others (9.05% to 9.84%) [20].

The second is growing conditions. The protein content varies with location. Varieties from Ayacucho had stable protein levels (around 9.45 g/100 g), whereas those from Huánuco and Huancayo ranged from 5.95 to 11.55 g/100 g [2]. Soil type, altitude, temperature, and rainfall may influence protein accumulation. When the same varieties were grown in New Zealand, their origin did not affect protein content [20], suggesting that the environment interacts with genetics.

The third is farming practices. Moreover, traditional practices such as crop rotation, organic fertilisation and farmer selection of seed tubers may affect protein content [2]. In the study of 50 INIA varieties [2], the genotypes were grouped into three categories: low (5.60–6.65 g/100 g), medium (7.00–9.98 g/100 g), and high (10.07–11.55 g/100 g). Twenty percent of the varieties exceeded 10 g/100 g.

Ulluco protein includes six of the nine essential amino acids: lysine, tryptophan, valine, isoleucine, leucine, and threonine [7,10,12,34]. Although some morphotypes present marginal limitations in sulphur amino acids (methionine and cysteine) [6,21], this profile is comparable to or higher than that reported for oca and mashua [7,10,12,34].

### 4.2. Micronutrients: Key Vitamins and Minerals

Ulluco stands out for its vitamin C content, with concentrations ranging from 23 to 28 mg per 100 g of fresh weight [16,20,32], which acts synergistically with other compounds to improve iron absorption and boost the immune system. Additionally, it provides B-complex vitamins (thiamine, riboflavin, and niacin) and vitamin A, which are essential for dermatological health [54].

Potassium is the most abundant mineral, with levels of 4.12% in whole flours, suggesting benefits for the control of arterial hypertension [13]. Relevant concentrations of magnesium (1.22%), phosphorus (0.32%), and calcium (0.29%) have also been identified; the latter is up to four times higher than in conventional potato [3,13]. Iron is reported to be between 1.1 and 1.23 mg/100 g, with variations depending on the agroecosystem and genotype [8,54].

### 4.3. Secondary Metabolites Beyond Betalains

Beyond its characteristic pigmentation, the arsenal of secondary metabolites in *U. tuberosus* is diverse and functionally relevant [45]. Triterpenoid saponins, known as tuberosides, constitute one of the groups of greatest pharmacological interest [7,51].

#### 4.3.1. Structural Characterisation of Tuberosides

Three novel triterpenoid saponins have been identified in methanolic extracts of ulluco using two-dimensional nuclear magnetic resonance (2D-NMR) and fast atom bombardment mass spectrometry (FAB-MS) techniques: tuberosides A, B, and C. Tuberouside A is the sodium and choline salt of 28-O-[β-D-glucopyranosyl] 3-O-[β-D-glucopyranosyl-(1→4)-β-D-glucuronopyranosyl]oleanate, with an oleanolic acid aglycone. Tuberouside B shares the same aglycone but features a more complex oligosaccharide chain: the sodium or choline salt of 28-O-[β-D-glucopyranosyl] 3-O-[β-D-xylopyranosyl-(1→2)-{β-D-glucopyranosyl-(1→4)}-β-D-glucuronopyranosyl]oleanate. Tuberouside C differs from B in its aglycone, which corresponds to hederagenin (23-hydroxy-12-oleanene), where a methyl group has been substituted by a hydroxymethyl group at the C-23 position, while maintaining the same sugar sequence [51]. The presence of choline as the cation, a rare occurrence in plant saponins, could be linked to specific transport or bioactivity functions, and it has been suggested that these saponins account, at least partially, for the characteristic flavour neutrality of ulluco compared to other Andean crops [7,51].

#### 4.3.2. Hypoglycaemic Activity and Mechanisms of Action

The hypoglycaemic activity of the butanolic fraction of *U. tuberosus* has been validated in animal models: the intraperitoneal administration of 55 mg/kg of a dry butanolic extract to induced diabetic rats significantly reduced glycaemia from values greater than 399 mg/% to 95 mg/% after 8 h [7,51]. However, this effect was achieved through intraperitoneal administration, bypassing gastrointestinal digestion and first-pass metabolism. To date, no studies have confirmed whether tuberosides retain their hypoglycaemic activity after oral consumption in humans, as their stability and bioavailability in the gastrointestinal tract remain unexplored. As summarised in Table 3, the isolation of purified tuberosides from fresh tubers yields only milligram-scale quantities (on the order of 10^−4^ % of fresh weight) [51]. The effective intraperitoneal dose in rats (55 mg/kg) would correspond to several grams of purified saponins for an adult human, while current extraction yields are in the milligram range per kilogram of fresh tuber. This indicates that direct extraction from raw material is impractical without more efficient recovery technologies.

The high content of mucilages present in edible tubers acts directly on the gastrointestinal tract, stimulating intestinal transit and exerting a natural laxative activity [7]. Despite these findings, some limitations should be noted. Oral bioavailability of tuberosides has not been established, as evidence comes from intraperitoneal administration in rodents; human pharmacokinetic and first-pass metabolism studies are lacking [7,51]. The extraction of purified tuberoside requires multiple solvent partitions and chromatographic steps (Amberlite XAD-2, Sephadex LH-20, DCCC, and HPLC), which limits scalability and increases costs [51]. Betalain extraction faces similar challenges due to interference from the protein and mucilage matrix of the tuber, requiring solid-phase extraction with C18 or C8 cartridges or ion-exchange resins to obtain high-purity fractions [15]. These technical barriers must be addressed before considering large-scale industrial applications.

#### 4.3.3. Relevance in Traditional and Regenerative Medicine

The presence of these metabolites supports the ethnobotanical use of ulluco as an anti-inflammatory and healing agent [5,51]. Tuber extracts stimulate the proliferation of human dermal fibroblasts and procollagen production, properties attributed to flavonols (rutin, narcisine), saponins, and betalains [7,13]. Catechol, identified in the tuber skin, acts synergistically with tuberosides to promote scarless tissue regeneration, positioning ulluco as a strategic resource for regenerative medicine and the pharmaceutical industry [3,13].

In addition to their antioxidant and wound-healing properties, *U. tuberosus* aqueous extracts have demonstrated antimicrobial activity against *Escherichia coli* [7], broadening the functional relevance of its secondary metabolite pool beyond pigmentation and antioxidant capacity.

#### 4.3.4. Phenolic Profiling and Identification of Novel Flavonoids

A recent analytical study provided additional details on the phenolic composition of ulluco. Ultra-high-performance liquid chromatography coupled with electrospray ionisation tandem mass spectrometry (UHPLC-ESI-MS/MS) was used to identify flavonoids as the predominant phenolic group in ulluco, with rutin, kaempferol-3-rutinoside and isorhamnetin-3-rutinoside among the most abundant compounds [55]. This study also reported the presence of quercetin-3-glucoside in ulluco tubers, a compound not previously described in this species, with the highest concentration (7.3 µg/g) found in the purple variety. The principal component analysis of the phenolic profiles, which explained 85.4% of the total variance, indicated that genotypes with orange-yellow and purple-orange pigmentation exhibited higher total phenolic content and ferric-reducing antioxidant power than yellow varieties [55]. These observations complement the previously described betalain profile and provide a more comprehensive basis for the chemotaxonomic discrimination of ulluco genotypes.

### 4.4. Betalains and Antioxidant Capacity

The phytochemical profile of ulluco includes up to 32 betalains identified by LC-DAD-ESI-MS/MS [1,15], with rare compounds such as arginine-betaxanthin (previously reported only in *Gomphrena globosa* inflorescences), as well as gomphrenin III and isogomphrenin III, among them. In red varieties, betanin, isobetanin, and acylated derivatives such as phyllocactin and isophyllocactin predominate [1,15].

The antioxidant capacity of ulluco betalains ranges from 483 to 1524 µg Trolox equivalents per gram of fresh weight [6,22], with a strong linear correlation (R^2^ = 0.9988) between total betalain content and Folin–Ciocalteu reduction [6,15,21,22]. The magenta betacyanin fraction exhibits markedly higher radical scavenging activity (TEAC = 1387.52 µmol/g) than the yellow betaxanthin fraction (51.55 µmol/g), confirming that betanin is intrinsically more active than indicaxanthin [15]. In certain genotypes, however, the hydrophilic antioxidant capacity of yellow varieties does not correlate directly with total betaxanthin content, suggesting that their phenolic acid profile also contributes [6,22].

From a technological perspective, ulluco betalains remain chromatically stable across a pH range of 3 to 7. This makes them suitable as natural colourants in diverse food matrices, including dairy products, meat products, and baked goods, where they could replace synthetic alternatives [1,14,17]. Their main limitation is thermosensitivity. Betacyanin and betaxanthin content decreases significantly within 5 h at temperatures above 80 °C, with acidic conditions accelerating this degradation [17]. This thermal instability restricts their use in products requiring pasteurisation, cooking, or other thermal processes above 80 °C without prior stabilisation or encapsulation [17,56]. Some strategies, such as microencapsulation with modified starches or pH adjustment to acidic conditions, may help mitigate this issue; however, these approaches require further optimisation for each specific application. However, under refrigeration (4 °C), colour changes remain minimal [15,17,21]. In addition, most evidence on betalain bioactivity comes from in vitro assays; oral bioavailability and efficacy in humans have not been confirmed [7,15]. Without human studies, current regulatory frameworks in major markets cannot support health claims. Emerging technologies, such as refractance window drying or freeze-drying, help preserve their functional properties [6,17]. Notably, polyploidisation increases betacyanin yield; induced tetraploid lines could provide a non-transgenic route to higher pigment content for industrial applications [1].

Compared to Andean tubers that accumulate anthocyanins (mashua, oca, native potato), ulluco exhibits lower total hydrophilic antioxidant capacity. The hierarchy reported in ABTS assays is mashua ≥ oca ≥ native potato ≥ ulluco [6,22]. Yet, betalains offer distinct advantages: superior chromatic stability across pH 3–7, where anthocyanins tend to degrade, and specific bioactivities in tissue regeneration, fibroblast stimulation, and antitumour assays [1,6,17].

This anatomical specialisation is consistent with the taxonomic position of *U. tuberosus* within the order Caryophyllales, the only plant lineage capable of synthesising betalains, and reflects a chemical defence strategy against UV radiation and abiotic stress characteristic of high-altitude Andean environments [6]. The near-total confinement of polyphenols, flavonoids, and betalains to the periderm (Table 3) is best interpreted as an anatomically targeted defence system rather than an incidental distribution, with the starch- and protein-rich pulp reserved for primary metabolism and energy storage.

Table 3 summarises the main classes of secondary metabolites identified in *U. tuberosus*, together with their reported quantities and validated bioactivities, complementing the qualitative description provided in the preceding sections.

## 5. Processing and Preservation Technologies

The use of ulluco has expanded beyond fresh consumption as a fresh vegetable or in traditional stews. This shift is driven by the need to mitigate the high postharvest losses resulting from its high water activity (83–90%) and perishability [8,10,41]. Current research focuses on the engineering of food matrices to stabilise their bioactive components and enhance the techno-functional properties of their starch using emerging technologies.

Table 4 summarises the main processing and preservation technologies discussed in this section, together with their principal application and current validation scale.

### 5.1. Matrix Challenges and Bioactive Stability

The ulluco processing faces challenges related to the stability of its betalains against severe thermal treatments. betalains are thermosensitive, with degradation accelerating above 80 °C. Acidified extracts (pH 4) retain the red hue better during storage [6,17]. The mucilage content varies between varieties, with 4.78% in *Puka Lisas* and 3.41% in *Papa Lisas* [12], complicating drying due to its high water retention capacity. Nevertheless, it possesses functional properties such as a stabiliser and potential wound healing and gastric protective effects [7,12,54].

### 5.2. Advanced Dehydration Technologies

Refractance window (RW) technology is superior to convective drying for obtaining ulluco slices. By using hot water (~84 °C) circulating under a plastic film transparent to infrared radiation, the process reduces moisture below 14% in approximately 51 min, minimising the thermal degradation of betalains in the *Puka Lisa* morphotype and preserving luminosity in the *Quello Lisa* [21]. Despite these advantages, some limitations should be noted. To ensure uniform drying kinetics, refractance window drying requires precise control of slice thickness (2.25–2.45 mm), as variations alter dehydration rates [21]. The physical deformation of slices due to cell shrinkage during processing also complicates morphological standardisation, which is required for automated packaging systems [21].

Traditional Andean processing (lingli or chullcce) constitutes a primitive form of freeze drying through nocturnal freezing and diurnal solar drying, which reduces moisture and concentrates proteins, fibre, and minerals, albeit with mucilage loss and colour changes due to oxidation [6,12]. In contrast, controlled laboratory freeze drying better preserves the protein profile, enabling the identification of high-protein variants useful for food security [2].

### 5.3. Engineering Starches and Edible Films

Compared to potato starch, Ulluco starch forms clear gels with less tendency towards retrogradation, making it suitable for refrigerated products [28,31]. Annealing treatment, a heat treatment in excess water, reorganises the crystalline structure without destroying it, increasing the relative crystallinity from 7.5% to 10.5% and the gelatinisation temperature, which improves thermal stability and reduces swelling [44,45].

Edible films plasticised with glycerol and chitosan have been developed, exhibiting good water vapour barrier properties (5.6 × 10^−11^ g/m·s·Pa) and high elasticity [27,57,59]. AFM analysis of these biofilms revealed a smooth, granular topography with nanoscale phase segregation, which facilitates oral disintegration and makes them candidates for drug delivery or oral hygiene sheets [58]. However, industrial scalability faces challenges. Batch-to-batch standardisation is difficult due to the biopolymer’s thermodynamic sensitivity. Nanoscale irregularities and microcracks have been observed in film topography, which compromise barrier properties such as water vapour permeability [44,58]. Achieving consistent morphology requires precise balancing of starch concentration, plasticiser content and gelatinisation conditions, a combination that is difficult to replicate on a large scale [27,44]. Additionally, esterification with octenyl succinic anhydride (OSA) confers amphiphilic properties to the starch, achieving encapsulation efficiencies greater than 80% for phenolic extracts from purple mashua via spray drying [56].

### 5.4. Extrusion Technologies and 3G Food Matrix Development Potential

Extrusion cooking technology for third-generation (3G) snacks has been applied to other Andean tubers such as oca and mashua, successfully modelling expansion kinetics and microstructure [32]. These studies show that extrusion partially degrades pigments, although the final product maintains acceptable phenolic content and antioxidant capacity under controlled conditions [61]. For ulluco, documented developments remain at the first-generation level, with extrusion into expandable pellets or 3G snacks not yet explored [10,12,21]. The partial substitution of wheat flour with ulluco flour in breadmaking improves the nutritional profile without negatively affecting sensory acceptability [4].

### 5.5. Biotechnology and Functional Applications

Ulluco starch gels allow for the construction of complex 3D structures with good resolution and dimensional stability, opening up prospects for personalised nutrition through food printing [36]. Nevertheless, several bottlenecks limit industrial translation. The rheological window for operability is narrow: starch concentrations above 12% cause nozzle clogging, whereas concentrations below 8% result in structural collapse [36]. Achieving high resolution requires fine nozzles, which require precise pressure control to avoid under-extrusion or material accumulation [60]. Moreover, natural variability in starch properties across regions, phenotypes, and seasons complicates consistency between batches, representing a major challenge for quality assurance at the industrial scale [62]. Starch has also been used as an adjunct in artisanal Ale-type beer (substituting up to 10–30% of malt), generating a beverage with acceptable physicochemical and sensory characteristics (pH 3.52 and 3.7% alcohol) [25], and as a natural texturiser in low-fat yoghurts [4]. Pharmacologically, ethanolic extracts have shown wound-healing activity in animal models, promoting collagen regeneration without dermal toxicity [7,54].

## 6. Applications in Modern Food Industry

The revaluation of *U. tuberosus* has transcended its traditional role as a subsistence crop to position it as an input for food-product innovation [7]. Its chemical versatility, focused on the quality of its starches and the uniqueness of its betalains, enables the development of products aligned with the global trends of “clean label” [3,16,45].

### 6.1. New Products: Snacks, Beverages, Colourants, and Functional Ingredients

In the snack food industry, ulluco has been considered a potential raw material for dehydration and extrusion processes. Some developments, such as dehydrated snacks of the “Olluchips” type, have demonstrated acceptable sensory acceptance and a moderate retention of phenolic compounds [10,21]. Compared to potato, ulluco may require fewer additives to maintain its nutritional value after dehydration and represents an option with low-fat content and the presence of betalains [7,10]. In addition to its direct food applications, ulluco by-products have also been explored as potential functional food ingredients.

Its use in fermented beverages has also been explored. For instance, an artisan Ale beer was produced incorporating 10% ulluco starch, with technically viable results and favourable sensory acceptance [25]. Likewise, its fermentable carbohydrates have been evaluated for bioethanol production [41].

The extraction of natural colourants from ulluco is another area of interest. As previously mentioned, betalains maintain stability across pH 3–7, making them suitable for use in products such as yoghurts, low-fat dairy products, and baked goods as an alternative to certain synthetic colourants [7,17,63].

### 6.2. Integration in Haute Cuisine and the Processed Food Industry

In the haute cuisine of Bolivia, Peru, and Colombia, some chefs have started to appreciate the crunchy texture that ulluco retains after cooking, a quality that distinguishes it from other more mealy potatoes [1,20]. In certain restaurants, its use is being explored through techniques such as pickling, the reduction of its juices to prepare vinaigrettes, or the utilisation of its mucilage as an emulsifier. Its earthy flavour, when combined with acidic ingredients, can bring a certain complexity to dishes [11].

Furthermore, in the field of food science and technology, ulluco has sparked interest due to its potential industrial applications. For instance, its gels have been assessed as suitable materials for 3D food printing, allowing for experimentation with controlled textures [36]. The production of edible biopolymer films from its starch, which has an amylose content of up to 35%, has also been studied with the aim of prolonging the shelf life of other products and offering an alternative to conventional plastics [27,57,59]. Additionally, ulluco flour has been used to enrich bread and other baked goods, improving their protein profile with amino acids such as lysine and tryptophan, which can be relevant for food security in some regions [2,4].

Finally, interest in the tuber is not limited to food. Its potential uses in avant-garde gastronomy, where some chefs have worked with its texture and pigments [11]. These initiatives point towards a more comprehensive utilisation of the resource, in line with some principles of the circular economy.

### 6.3. By-Product Valorisation and Circular Economy Approaches

The processing of ulluco generates organic residues, including peels, stems, and leaves. Studies have indicated that these by-products contain bioactive compounds and nutrients that could be recovered for food or biotechnological applications.

On a dry basis, leaves can contain up to 12% protein [10,47]. They also provide iron, vitamin A, and mucilage. The leaves are occasionally consumed as a leafy vegetable in salads, stews, and soups in the Andean regions of Peru and Colombia [10]. Additional studies are needed to validate these findings across different cultivars and growing conditions and to assess consumer acceptance and food safety.

The peel contains a higher phenolic content than the pulp. Histochemical analysis confirmed the presence of phenolics, flavonoids, and triterpenes in the epidermal and subepidermal tissues of the tuber skin [30]. Betalain content follows the same pattern, with substantially higher concentrations in the peel than in the pulp, as shown in Table 3. Stems contain levels of betalain comparable to those found in tubers, with dopamine-betaxanthin and betanin as the major pigments [1].

Peel extracts from ulluco promote biofilm formation in the probiotic strain *Lactobacillus acidophilus* La-14 in a dose-dependent manner [30]. Organic waste from ulluco processing has also been used as a substrate for microbial fuel cells, achieving a voltage of 0.99 V and a power density of 373.8 mW/cm^2^, with a 94% reduction in chemical oxygen demand [59]. These findings suggest the potential applications of ulluco by-products in symbiotic formulations, bioenergy generation, and integrated biorefinery approaches.

## 7. Discussion

### 7.1. The Ulluco Paradox: Outstanding Potential, Persistent Marginalisation

The information gathered in this review reveals a paradoxical situation concerning ulluco. On the one hand, the tuber exhibits a protein content comparable to that of other Andean crops such as oca and mashua, reaching up to 15.7 g per 100 g of dry matter [12]. Furthermore, it can grow in adverse conditions where few food crops manage to thrive altitudes above 4000 m a.s.l., soils with low organic matter, and exposure to frost or drought [3,19,23,25]. From an agronomic standpoint, it is one of the most resilient food crops in South America. However, its presence in international markets remains very limited, the scientific output related to it is sparse compared to other tubers, and its industrial processing chain is virtually non-existent [11,20,49].

This situation does not appear to be coincidental. Rather, it could reflect a structural difficulty that food science has not addressed with sufficient depth: the gap between laboratory-generated knowledge and the institutional, regulatory, and agro-industrial conditions necessary to promote an underutilised crop [11]. Unlike quinoa, whose growth in global markets was accompanied by nutritional research, institutional recognition, and export infrastructure development [64], ulluco has not received similar support. As a result, part of the accumulated knowledge about its properties is scattered in university repositories and postgraduate theses, failing to achieve a prominent presence in high-impact scientific journals or in the public policy agendas focused on investment in food systems [7,25]. To overcome this hurdle, conducting additional research would not be sufficient; it would also be necessary to establish more effective bridges between genomics, food technology, and international trade regulations.

Nevertheless, it would be inaccurate to attribute ulluco’s limited diffusion solely to institutional factors. Technical limitations also exist and should be acknowledged. Its high moisture content, which ranges between 80% and 85%, and the delicacy of its skin complicate post-harvest handling and can lead to significant losses if proper refrigeration infrastructure is not available [10,12]. Moreover, its dependence on a short photoperiod restricts its cultivation to certain latitudes, limiting production outside the Andean region without resorting to light cycle manipulation techniques [23,47]. Furthermore, as it is primarily propagated using seed tubers, there is an accumulated phytosanitary risk, since successive vegetative multiplication can facilitate the transmission of viruses that affect crop yield and quality [9,26,46]. These difficulties are not insurmountable but addressing them would require sustained investments in clean seed programmes, cold chains, and genetic improvement, efforts that can hardly be assumed solely by the academic sector.

**Figure 5:**
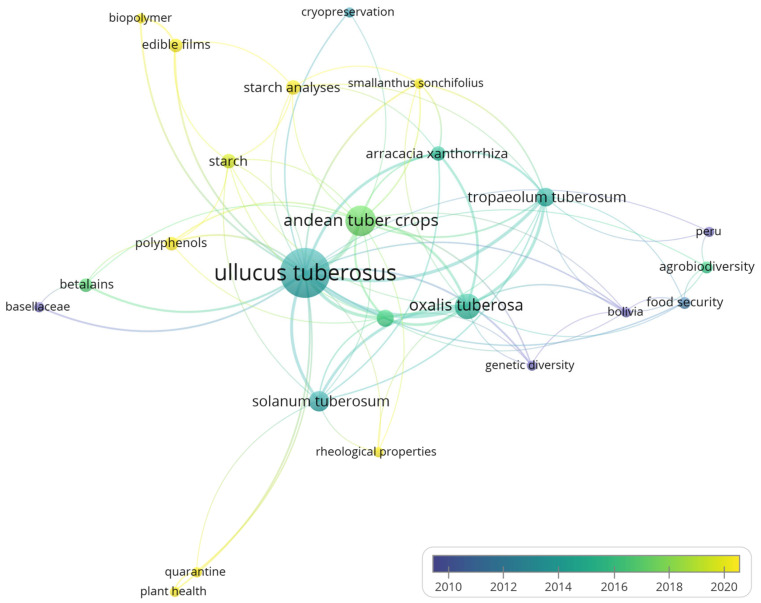
Overlay visualisation network map of keyword co-occurrences mapping the limited global literature on the subject. Data were retrieved from the Scopus database (N = 128 documents, unrestricted timescale) using the query “*Ullucus tuberosus*” OR ulluco OR olluco and analysed via VOSviewer (v.1.6.20). Node size represents keyword frequency within this small corpus, tracking the shift from early conservation topics (purple/blue) toward a tight, recently emerging cluster of agroindustrial applications (yellow, ~2018–2022).

This paradox is evident in the available scientific literature. A systematic search of the Scopus database using the comprehensive query (“*Ullucus tuberosus*” OR ulluco OR olluco), with no time restrictions, yielded a remarkably low 128 studies as of May 2026. This scarcity confirms that the international scientific visibility of this Andean tuber remains critical. Furthermore, when analysing this limited corpus through VOSviewer (version 1.6.20), the chronological distribution in Figure 5 reveals why the literature feels so fragmented. The historical core between 2010 and 2016 was strictly confined to foundational agronomic topics, agrobiodiversity, and regional food security, linking *U. tuberosus* to other traditional crops like *O. tuberosa*. It is only in the most recent window between 2018 and 2020 that a small, emerging cluster has begun exploring post-harvest technological integration, such as starch extraction, polyphenols, and edible films, as illustrated by the peripheral yellow nodes in Figure 5. This low publication volume underscores an urgent research gap, highlighting a critical need to transition from basic local conservation data into broader, well-documented agroindustrial and technological international debates.

### 7.2. Betalains as a Competitive Differentiating Advantage: Complementarity, Not Competition

An aspect that merits attention in the comparative literature on Andean tubers is the tendency to evaluate their bioactive profiles in terms of hierarchy, giving greater relevance to those with the highest antioxidant capacity. Under this criterion, ulluco’s antioxidant capacity is markedly lower than that of mashua and oca (Table 1), which might make it seem less promising. This interpretation, although numerically grounded, could overlook a distinctive feature of ulluco: the type of bioactive compounds it synthesises.

The relevant difference lies not so much in the quantity of antioxidants as in their nature. While mashua, oca, and many native potatoes produce anthocyanins, flavonoid pigments with known antioxidant capacity but sensitive to changes in pH, temperature, and light [1,37], ulluco is the only member of the Basellaceae family that synthesises betalains. These nitrogenous pigments maintain their chromatic stability in a pH range of 3–7, which covers most food matrices, from fermented dairy products to baked goods [1,17,22]. This characteristic has practical implications in the food industry, where synthetic colourants are still widely used, partly because natural anthocyanin-based alternatives often exhibit instability during processing. In this context, ulluco betalains could offer a different option [1].

Beyond colour, the bioactivity profile of ulluco betalains presents certain characteristics. For example, the magenta betacyanin fraction shows markedly higher radical scavenging activity than the yellow betaxanthin fraction [15]. Studies have shown that certain ulluco extracts can stimulate the proliferation of dermal fibroblasts, a process related to tissue regeneration [5], and that its betalain pigments have shown some chemopreventive potential in preliminary studies with cancer cell lines [3,6]. These effects appear to operate through pathways distinct from those associated with anthocyanins, suggesting that ulluco and other Andean tuber extracts should be considered more as complements than as substitutes in the development of nutraceutical products.

The presence in ulluco of uncommon compounds, such as arginine-betaxanthin, previously identified only in inflorescences of *Gomphrena globosa*, and the recently described Gomphenin III and Isogomphenin III, adds distinctive elements to its phytochemical profile and could be useful for authenticity certification in specialised markets [1,15]. Triterpenoid saponins, known as tuberosides A, B, and C, whose hypoglycaemic activity has been explored in animal models and whose structure, which includes a choline cation, suggests possible functions in biological transport, are also present [51]. Collectively, these background data indicate that the interest in ulluco is not limited to its antioxidant capacity, and that its pharmacological or functional potential merits evaluation with criteria that consider its chemical specificity.

### 7.3. The Potato as an Industrial Benchmark: Validated Applications in Solanum tuberosum L. and Unexplored Applications in Ullucus tuberosus

The potato (*S. tuberosum*) serves as a benchmark in the industrial processing of starchy tubers, possessing a developed value chain that includes frozen products, dehydrated flakes, native and modified starches, gluten-free flours, fermentation substrates, and bioactive extracts. Table 5 presents ten areas of industrial application in which potato processing is commercially established and compares them with the current development status of equivalent applications in ulluco. This comparison suggests that, in cases where ulluco has been evaluated, its technical behaviour is, in some aspects, comparable to that of the potato. In areas where studies have not yet been conducted, the available compositional information would allow its viability to be considered at a minimum. The differences between both tubers do not appear to derive so much from intrinsic limitations of ulluco, but rather from the lower level of investment in research oriented towards its potential industrial applications.

From the analysis presented in Table 5, some useful elements can be identified to guide future research. On the one hand, there are applications in which ulluco shows a technical profile close to that of potato, such as gluten-free flours, biodegradable films, and dehydrated snacks. In these cases, moving the laboratory results to a pilot-scale might be sufficient to evaluate their feasibility as commercial ingredients. These are probably the options with the lowest investment requirement and the greatest short-term development potential.

On the other hand, ulluco presents characteristics that do not have a direct equivalent in potato, such as its betalains for use as natural colourants or its possible application in cosmeceutical formulations aimed at wound healing. In these niches, the specificity of ulluco could be advantageous because it would not directly compete with potato-derived products. Moreover, the technology for isolating ulluco proteins has not yet been developed, which opens another line of exploration. Collectively, these areas could merit priority attention in a potential research agenda aimed at diversifying the uses of the crop.

### 7.4. Scientific Gaps as a Research Agenda: A Critical Evaluation

**Figure 6:**
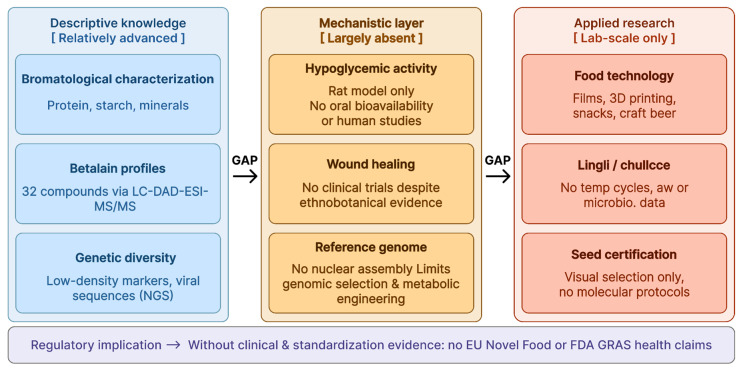
Scientific knowledge gaps in *Ullucus tuberosus* research across three evidence tiers: descriptive, mechanistic, and applied.

A limitation in the current knowledge on *U. tuberosus* is not so much a lack of information but rather its uneven distribution throughout the research process. In the descriptive field, the bromatological characterisation of various morphotypes is relatively advanced, with quantitative data on protein, starch, betalains, and mineral content available for accessions from different geographical origins [8,12,16]. At the applied end, preliminary studies have explored the technical feasibility of ulluco in edible biopackaging, inks for 3D food printing, dehydrated snacks, or adjuncts for craft beer, all conducted at the laboratory-scale [25,27,32,36,45]. Less explored is the intermediate stage: mechanistic studies, in vivo trials, or clinical investigations that would allow the level of evidence required by current regulations to support potential health claims. This uneven distribution throughout the research process is summarised in Figure 6.

For instance, the hypoglycaemic activity of butanolic extracts rich in saponins has been observed in a study on rats, through intraperitoneal administration of 55 mg/kg, resulting in a reduction in blood glucose levels from over 399 mg/% to 95 mg/% in eight hours [51]. This is an interesting preliminary result, but data on oral bioavailability, effective doses in dietary contexts, or human glycaemic response studies are still lacking. Similarly, the effects of ulluco extracts or compounds such as catechol present in its peel on fibroblast proliferation and procollagen synthesis [5,13] have not been evaluated in controlled clinical trials, despite the traditional use of the tuber as a wound-healing agent being documented in ethnobotanical literature [4,5,51]. This gap between traditional knowledge and clinical validation has practical implications, as without studies meeting regulatory standards, it is not possible to support health claims in European or American markets.

At the genomic level, research has mainly focused on identifying viral sequences using high-throughput sequencing techniques [46] and low-density markers for diversity studies [19,49]. However, the absence of a complete assembly of the nuclear genome of *U. tuberosus* limits the development of more efficient breeding strategies. The selection of heat-tolerant or virus-resistant varieties without a reference genome depends on phenotypic methods or traditional markers, which are generally slower and less accurate than the genomic selection approaches applied in other crops. This limitation is more relevant in the context of the climate projections in Section 7.5. The availability of a reference genome could also facilitate metabolic engineering studies and contribute to the conservation of crop diversity.

Another area where gaps are observed is the standardisation of traditional processing techniques. Lingli or chullcce, an ancestral method of freeze drying and solar drying practised in Andean communities, allows obtaining a product with a higher concentration of protein, fibre, and minerals [6,12]. Nevertheless, this process has not been documented in the scientific literature with the level of technical detail, such as temperature cycles, water activity monitoring, or microbiological safety parameters, that would be necessary for its eventual industrial scaling or to comply with the requirements of regulations such as the European Union Novel Foods Regulation or the United States Food and Drug Administration (FDA) Generally Recognized as Safe (GRAS) framework.

Finally, other relevant operational gaps persist. Seed tuber certification schemes continue to rely on visual selection by farmers, without having standardised molecular protocols [9]. Likewise, although progress has been made in the quantitative characterisation of protein variability between morphotypes, studies associating specific functions of low-molecular-weight proteins with agronomic traits of interest are not yet available [2].

### 7.5. Projections Under Climate Change and Crop Resilience

Despite its recognised capacity to grow in adverse environmental conditions, *U. tuberosus* could also be affected by climate change. Some projections carried out with the AquaCrop model indicate that the thermal stress expected by the year 2100 could represent a threat to its cultivation at high altitudes [16], precisely the environment where ulluco has been traditionally grown and where it has comparative advantages over other crops.

The main risk factor would be associated with the displacement of the optimal thermal ranges for the crop. Ulluco requires temperatures between 8 and 14 °C for adequate development [9,12]; a sustained increase in temperature could reduce the effective altitude for its cultivation, decreasing the available agricultural area in the central Andes and displacing production towards higher zones, where soils are usually less fertile and radiation is more intense [16,18]. This scenario could be particularly relevant in Bolivia and the high Andean regions of southern Peru, where the crop is part of subsistence economies with limited technological adaptation capacity.

In parallel, the increase in water variability, with more frequent and intense droughts alongside extreme precipitation, could affect the tuberisation process, which is sensitive both to water deficit during the thickening phase and to excess moisture in soils with limited drainage [16,23]. In this context, the convenience of implementing water harvesting and storage strategies has been highlighted, as well as strengthening crop rotation systems adapted to high Andean agroecosystems [16]. These measures would also be linked to clean seed programmes, since a certified seed stock could have its potential reduced if the production systems that support it are vulnerable to water variability [9].

Given this outlook, molecular breeding aimed at heat tolerance and adaptation to longer photoperiods could constitute a relevant line of work, both to reduce climatic risks and to expand potential cultivation areas towards currently marginal zones. The tetraploid lines that have already been identified for producing higher concentrations of betalains could be considered in breeding programmes aimed at optimising pigment content [1]. These strategies would not necessarily require transgenic genetic modification; marker-assisted selection, eventually supported by a reference genome like the one suggested in Section 7.4, could be sufficient in advancing these objectives within agronomically viable timescales.

### 7.6. Towards Industrialisation: A Roadmap for the 21st Century

The information gathered in this review suggests that the primary challenge for the industrial development of ulluco lies not in a lack of knowledge about its functional properties but rather in the absence of mechanisms that allow this knowledge to be translated into concrete applications. Based on the gaps identified in the literature, the following research priorities are proposed with a hierarchical classification according to their relevance for industrial translation, as summarised in Table 6.

These priorities are not necessarily sequential; advances in genomics can accelerate phytosanitary programmes, and clinically validated preparations are essential for processing standardisation. A transversal governance dimension linked to the Nagoya Protocol, which establishes the framework to guarantee equitable returns to the Andean communities that have maintained and diversified the crop, is underlying all of them [11].

The use of ulluco by-products in animal nutrition has also been proposed as a potential application. Phenolic compounds and betalains present in Basellaceae species have been suggested to have antimicrobial or anti-inflammatory properties relevant to livestock feed, although this proposal is based on evidence from aquaculture and rodent studies; trials in poultry or swine have not been conducted [83].

In summary, *U. tuberosus* has a set of properties that could support its transition from an underutilised crop to a functional ingredient of interest. For this transition to occur, descriptive research would need to be complemented by efforts aimed at generating clinical evidence, phytosanitary clean-up, process standardisation, and genomic tool development. In this task, food science could play a role not only in generating knowledge but also in identifying the conditions that allow its application in specific productive and regulatory contexts [11,16].

### 7.7. Regulatory Considerations for Industrialisation

#### 7.7.1. Phytosanitary Restrictions in the European Union

Under Commission Implementing Regulation (EU) 2018/2019, *U. tuberosus* tubers are provisionally classified as “high-risk plants”, which restricts their introduction into the EU until a scientific opinion is issued [9]. The EFSA conducted a Commodity Risk Assessment in response to the dossier submitted by the Peruvian National Plant Protection Organisation (SENASA). The assessment identified five quarantine pests of concern: the nematodes *Nacobbus aberrans* and *Atalodera andina*, the viruses APLV and PVT, and the ulluco weevil (*Amathynetoides nitidiventris*) [9]. Although ulluco belongs to Basellaceae and not Solanaceae, its frequent rotation with potato crops indicates that it can act as a vector for shared quarantine pests [9,64].

SENASA has established operational procedures for export to address these restrictions. These include brushing and washing tubers to remove soil, visual inspection of 2% of export batches, and controlled transport conditions (2 °C, 90–95% RH) [9,64]. Unregulated trade through digital platforms poses an additional concern. In the UK, exotic viruses have been detected in plants grown from informally imported tubers, indicating that visual inspection alone cannot reliably detect asymptomatic or latent pathogens [9,46].

#### 7.7.2. Classification of the Processed Derivatives

The regulatory pathway for ulluco-derived products intended for the EU market depends on the degree of processing. Minimally processed products such as flours and flakes could follow the Traditional Food from a Third Country (TFTC) pathway under Regulation (EU) 2015/2283. This pathway requires notification to the European Commission with evidence of traditional use, without the need for a comprehensive safety evaluation if no objections are raised [84].

Concentrated extracts, such as betalain pigments or isolated tuberosides, would require a different approach. If the European Commission or EFSA raises safety objections, the applicant must submit a comprehensive dossier under Article 16 of Regulation (EU) 2015/2283, including data on composition, toxicology, allergenicity, and anticipated exposure [84]. The low extraction yields reported for tuberosides (1.3 × 10^−4^ to 5.3 × 10^−4^ % of fresh weight) [51] and the absence of oral bioavailability data should be addressed in any such application.

#### 7.7.3. Regulatory Context in the Andean Region

Legislation and state policies for ulluco production vary among Andean nations, with inconsistencies and limited implementation [3]. A unified regional policy has been suggested to strengthen institutional knowledge exchange and ensure the protection of high-value genetic resources under effective agricultural biodiversity conservation laws [3].

In Peru, Law No. 30021 and Supreme Decree No. 017-2017-SA regulate the processing of ulluco into products such as dehydrated snacks, which require warning octagons on ultra-processed foods. Dehydrated ulluco products processed without added saturated fats may align with the guidelines for reduced-warning-label claims [10].

#### 7.7.4. Implications for Industrialisation

This regulatory framework has several implications for ulluco industrialisation. Processing in the country of origin may be advantageous because value-added products such as flours and extracts are not subject to the same phytosanitary restrictions as fresh tubers [9]. The TFTC pathway appears feasible for minimally processed products with adequate documentation of traditional use [84]. For extracts and isolated compounds, the Novel Food pathway in the EU requires more extensive safety data and would likely require longer-term investment [84].

In the United States, no specific FDA determination exists for ulluco, but any ulluco-derived product would need to meet the GRAS standard [85]. Because ulluco has a long history of safe consumption in the Andes, its evaluation would focus on demonstrating that its composition is comparable to known safe foods [86]. This would include identifying any naturally occurring compounds of concern and estimating how much would be consumed [85]. Complex toxicokinetic studies are often not practical for whole foods like ulluco [86], but targeted safety evaluations and monitoring after market entry can address any remaining concerns [85,86]. As outlined in Table 6, these distinctions are relevant for prioritising research and development efforts.

## 8. Conclusions

The information presented in this review indicates that *U. tuberosus* possesses characteristics that could be of interest to the food and biotechnology industries. From a nutritional standpoint, ulluco exhibits a protein content that can reach up to 15.7% on a dry basis and includes six of the nine essential amino acids for human nutrition. However, the most distinctive feature of ulluco is its betalain profile: pigments that maintain their chromatic stability within a pH range of 3–7 and exhibit different properties from the anthocyanins present in other Andean tubers. This characteristic opens up the possibility of developing natural food colourants with potential applications in processed foods. Furthermore, available preclinical studies have reported effects such as dermal fibroblast stimulation in cell models and blood glucose reduction in experimental animals, attributed to compounds like tuberosides. These are preliminary results that provide a mechanistic basis, but they need to be supplemented with human clinical trials to support any eventual therapeutic applications. From a food science and technology perspective, ulluco has demonstrated versatility in various laboratory-scale applications. Its starch, which constitutes around 65% of its dry matter, can be used in edible film formulations, 3D food printing matrices, dehydrated snacks, fermented beverages, and wholemeal flours. However, further studies are needed to assess its pilot-scale performance and acceptance in specific markets.

Despite these advantages, several industrial bottlenecks restrict commercialisation. Betalain thermosensitivity above 80 °C limits its use without prior encapsulation. Purified betalain and tuberoside extraction requires complex chromatographic protocols, and oral bioavailability in humans has not been established. Processing technologies have been validated only at the laboratory scale, with challenges in standardisation and narrow rheological windows. The lack of a complete nuclear genome assembly limits marker-assisted breeding for heat tolerance and virus resistance. Addressing these bottlenecks requires a coordinated agenda that integrates phytosanitary certification, pilot-scale validation, clinical studies, and genomic tools, while ensuring that returns reach the Andean communities that have conserved this crop.

## Figures and Tables

**Table 1:** Comparative table of the nutritional and functional composition and industrial potential of ulluco compared to other Andean tubers.

Characteristic	Ulluco (*Ullucus* *tuberosus*)	Oca (*Oxalis* *tuberosa*)	Mashua (*Tropaeolum tuberosum*)	Native Potato (*Solanum* *tuberosum* L.)	Yacon (*Smallanthus sonchifolius*)
Proteins (g/100 g dw)	5.60–15.70	3.0–15.9	6.9–15.7	1.60–9.54	4.2 (var. INIAP-ECU-1247)
Total Phenols	0.41–0.77 mg GAE/g fw	0.71–1.32 mg GAE/g fw	450 ± 7 mg GAE/100g dw; up to 337 mg GAE/100g fw (~0.9–1.1 mg GAE/g fw)	0.64–2.32 mg GAE/g fw	29.65–33.8 mg GAE/g dw (~3.0–5.1 mg GAE/g fw)
Principal Pigment	Betalains (betacyanins, betaxanthins; phyllocactin, isophyllocactin, gomphrenin)	Anthocyanins (glucosylated derivatives of malvidin, petunidin, peonidin) and carotenoids	Anthocyanins (di-/triglucosides of delphinidin, cyanidin) and carotenoids	Anthocyanins (purple/red ecotypes) and carotenoids (violaxanthin, lutein in yellow ecotypes)	Variable phenotypes (white to purple); no industrial use as a colourant
Antioxidant Capacity	ABTS: 483–1524 μg TE/g fw. DPPH: 2.4 mM TE/100g dw	ABTS: 1637–4771 μg TE/g fw. FRAP: 420.42 μM TE (peel)	ORAC: 273–379 μmol TE/g DM. ABTS: 955–9800 μg TE/g fw. DPPH: 6.0 mM TE/100g dw	ABTS: 860–3780 μg TE/g fw. ORAC: ~13.1 μmol TE/g fw	DPPH: 4.4 mM TE/100g dw. ABTS: 111.45 μM TE (pulp)
Total Carbohydrates (% dw)	64.96–84.2	75.4–88.8	69.7–79.5	~80.0–85.0	~85.0
Pure Starch (% dw)	52.0–70.5	~42.17	56.22–66.0	66.0–80.0	0.0 (accumulates FOS)
Key Bioactive Compounds	Betalains, vitamin C, mucilages (hydrocolloid polysaccharides), flavonoids (rutin, narcissin, kaempferol)	Phenolic acids (vanillic, caffeic, cinnamic), ocatin (antimicrobial proteins), FOS, vitamin C	Isothiocyanates, glucosinolates, vitamin C, essential amino acids, proanthocyanidins	Chlorogenic acid, quercetin, lutein, vitamin C (22.2–121 mg/100g dw)	FOS: kestose, nystose (40–70% dw); phenolic acids (chlorogenic, caffeic)
Main Industrial Potential	Biodegradable films, natural colourants (stable pH 3–7), thickener/gelling agent, and prebiotic ingredients	Gluten-free flours, baked foam trays, oleogels, drug encapsulation	Bio-inks for 3D food printing, cosmetics (anti-ageing), 3G extruded snacks	Bioethanol, biofilms, phenol extraction from peel, expanded snacks	Prebiotic syrups for diabetics, microbiota modulation, fermented ciders
References	[1,4,6,7,10,13,17,21,22,28,29,30]	[6,12,14,22,31,32,33]	[22,29,32,34,35,36,37,38,39,40]	[20,22,29,32,40,41,42,43]	[3,11,14,29]

Note: fw = fresh weight; dw = dry weight; DM = dry matter; TE = Trolox equivalent; GAE = gallic acid equivalent; FOS = fructooligosaccharides; ORAC = oxygen radical absorbance capacity; ABTS = 2,2′-azinobis-(3-ethylbenzothiazoline-6-sulfonic acid) assay; DPPH = 2,2-diphenyl-1-picrylhydrazyl assay; FRAP = ferric reducing antioxidant power assay; mg = milligram; μg = microgram; g = gram; mM = millimoles per litre; μM = micromoles per litre; INIA = Instituto Nacional de Innovación Agraria (Peru). The term “Native potato” under the taxon Solanum tuberosum L. encompasses the groups of Andean cultivars with high phytochemical diversity, historically recognised as S. tuberosum ssp. andigenum and S. phureja.

**Table 2:** The 32 betalains identified in *U. tuberosus* via LC-DAD-ESI-MS/MS.

Betacyanins (12 Compounds)
Betanin; Isobetanin; Gomphrenin I; Phyllocactin; Betanidin; Isogomphrenin I; Isophyllocactin; Isobetanidin; Betanidin-monoferuloyl-5-O-β-diglucoside; Isobetanidin-monoferuloyl-5-O-β-diglucoside; Lampranthin II; Isolampranthin II
**Betaxanthins (20 compounds)**
Histidine-betaxanthin; asparagine-betaxanthin; serine-betaxanthin; arginine-betaxanthin; glutamine-betaxanthin; aspartic acid-betaxanthin; lysine-betaxanthin; threonine-betaxanthin; glutamic acid-betaxanthin; proline-betaxanthin; dopa-betaxanthin; tyrosine-betaxanthin; dopamine-betaxanthin; methionine-betaxanthin; valine-betaxanthin; tyramine-betaxanthin; 3-methoxytyramine-betaxanthin; isoleucine-betaxanthin; leucine-betaxanthin; phenylalanine-betaxanthin

**Table 3:** Secondary metabolites, reported quantities, and bioactivities of *U. tuberosus*.

Secondary Metabolite Class	Reported Quantity	Bioactivity
Betalains (betacyanins, betaxanthins; e.g., histidine-betaxanthin, arginine-betaxanthin, betanin, and iso-betanin)	Up to 66.3 µg/g in the tuber peel [15]; absent from the whole-tuber anthocyanin profile [6].	Antioxidant capacity and natural colourant potential [14].
Catechol-type polyphenols	23.91 mg GAE/g (red) and 21.86 mg GAE/g (yellow) in the peel, exceeding the pulp [14].	Concentrated in outer tissues (skin) [6].
Flavonoids (rutin, narcissin, and kaempferol derivatives)	16.57 mg CE/g in the peel of the red variety [14].	Kaempferol inhibits lipid peroxidation and scavenges superoxide radicals [6,21].
Triterpenoid saponins (tuberosides A, B, and C)	15 mg total isolated from 1.5 kg fresh tuber (1.3–5.3 × 10^−4^ %) [51].	Acute hypoglycaemic activity; IP 55 mg/kg reduced glycaemia from >399 to 95 mg/% in 8 h [51].
Complex extracts (polyphenols, alkaloids, tannins, and triterpenes)	Not quantified at the molecular level.	Wound-healing: increased collagenase activity (12%), procollagen, and dermal fibroblast proliferation [5].

**Table 4:** Processing and preservation technologies applied to *U. tuberosus*, with their validation scale.

Technology	Application	Scale of Validation	References
Acidification of the extract (pH adjustment)	Betalain colour stabilisation	Laboratory	[6,17]
Refractance Window (RW) drying	Dehydrated slices	Laboratory	[21]
Traditional freeze-drying method (lingli/chullcce)	Ancestral dehydrated product	Artisanal/traditional	[6,12]
Controlled laboratory freeze-drying	Protein-preserving dehydration	Laboratory	[2]
Starch annealing	Modified starch	Laboratory	[44,45]
Formation of edible film (glycerol/chitosan)	Packaging and drug delivery	Laboratory	[27,57,58,59]
OSA esterification + spray drying	Encapsulation of phenolic extract	Laboratory	[56]
Extrusion/thermal processing	Dehydrated snacks and instant flours (1 g)	Commercial	[10,12,21,32]
3D food printing	Personalised nutrition matrices	Laboratory	[36,60]

**Table 5:** Validation of industrial applications in *Solanum tuberosum* L. and their current state of development in *Ullucus tuberosus*, with justification of feasibility for translational investment.

No.	Application	Potato (*Solanum tuberosum* L.)	Ulluco (*Ullucus tuberosus*)	Research Gap
1	Gluten-free flour	Commercially validated. Potato flour and starch mitigate the absence of gluten networks due to their high swelling capacity and gas retention [65,66].	Preliminary evaluation of Andean bread and gluten-free products with 30–70% substitution shows favourable textural modifications and sensory acceptance [4,33].	Characterise dough rheology at industrial scale under mechanical stress.
2	Resistant starch (prebiotic)	Commercially developed. Clinical trials confirm gut microbiota modulation with 3.5 g/day [67].	Low native content due to high digestibility (80–94%) [7,35]. Annealing modification increases crystallinity (7.5% to 10.5%) [44,45].	Evaluate technological induction of RS3 (retrograded resistant starch) and in vivo prebiotic efficacy.
3	Biodegradable films	Industrially established. Films with bacterial cellulose or nanoparticles offer high barrier and resistance [68,69,70].	Laboratory-scale: edible films with permeability 5.6 × 10^−11^ g/m·s·Pa, high elasticity and nanometric topography [27,44,45,57].	Pilot-scale prototyping under GMP and shelf life characterisation.
4	Natural colourant	Pigmented varieties are processed for anthocyanins as colourants and antioxidants [71,72].	Unique: accumulates pH 3–7 stable betalains (not anthocyanins). Spray-dried encapsulation >80% [3,7,17,56].	Develop a commercially standardised extract. No direct competition with potato. High market potential.
5	Food 3D printing	Potato starch is recognised for its controllable rheological consistency [61,73].	8–12% gels exhibit pseudoplasticity with dominant elastic modulus (G’). Basic 3D structures [36].	Replicate at pilot scale and optimise printing parameters.
6	Extruded snacks	Fully industrialised. Optimised conditions: 14% moisture, 550 rpm, 170 °C [61,73,74,75,76,77].	Sensorially validated dehydrated snacks (Olluchips) [10,21]. Proposal for instant extruded flours with quinoa [12]. Favourable fat absorption index and hygroscopic profile [38].	Optimise the extrusion of pure ulluco flour. Feasible with existing infrastructure.
7	Fermented beverages	Starch and juice are used for anthocyanin-rich beers and probiotic beverages [72,78,79,80].	Ale-type beer with 10–30% malt substitution: pH 3.52, 3.7% alcohol, sensory acceptability [25]. Scarce reports in dairy products [63].	Sensory optimisation, scaling up, and betalain stability during fermentation.
8	Bioethanol production	Extensively studied and industrialised [78,79,80].	Only preliminary studies on fermentable carbohydrate yield [7,41]. No Q1 publications in biorefinery.	Unviable as an independent source; viable only in integrated biorefinery (colourant extraction + proteins + residue fermentation).
9	Cosmeceuticals (wound healing)	Glycoalkaloids and polyphenols from residues accelerate tissue regeneration [42,71].	In vitro: extracts induce fibroblast migration and upregulate the activity of procollagen, collagenase, and MMP-1 [5,7,29]. Documented ethnomedicinal use.	Formulate dermatological hydrogels and conduct Phase I clinical trials. No equivalent in potato.
10	Protein isolate	Patatin isolate commercially available globally: biological value comparable to egg, emulsifying properties [65,81,82].	Protein content: 5.60–15.7 g/100g dw; 20% of varieties >10g; 6 essential amino acids identified [2,34,44].	No studies on isoelectric precipitation extraction or techno-functional characterisation.

Note: The term “Potato” under the taxon *Solanum tuberosum* L. encompasses the Andean cultivar groups of high phytochemical diversity, historically recognised as *S. tuberosum* ssp. *andigenum* and *S. phureja*.

**Table 6:** Hierarchical classification of research priorities for *U. tuberosus* industrial translation.

Priority	Timeline	Actions
Short-term	1–3 years	Phytosanitary certification and clean seed programmes; standardisation of betalain extraction protocols; pilot-scale validation of dehydration technologies.
Medium-term	3–7 years	Pilot-scale validation of edible films, 3D printing, and dehydrated snacks; development of standardised processing methods for traditional products; and genomic characterisation using ISSR and RAPD.
Long-term	>7 years	Clinical validation through human intervention studies; complete nuclear genome assembly; heat tolerance and virus resistance marker-assisted selection.

## Data Availability

No new data were created or analyzed in this study. Data sharing is not applicable to this article.

## Abbreviations

The following abbreviations are used in this manuscript:

ABTS	2,2′-azino-bis(3-ethylbenzothiazoline-6-sulfonic acid)
DM	Dry Matter
DPPH	2,2-diphenyl-1-picrylhydrazyl
dw	Dry Weight
FDA	Food and Drug Administration (United States)
FOS	Fructooligosaccharides
GAE	Gallic Acid Equivalent
GRAS	Generally Recognized as Safe
INIA	National Institute of Agrarian Innovation (Peru)
LC-DAD	Liquid Chromatography with Diode Array Detector
ORAC	Oxygen Radical Absorbance Capacity
RAPD	Random Amplified Polymorphic DNA
SDS-PAGE	Sodium Dodecyl Sulfate Polyacrylamide Gel Electrophoresis
SENASA	National Plant Protection Organisation (Peru)
TE	Trolox Equivalents
TEAC	Trolox Equivalent Antioxidant Capacity
